# A new communication approach to encourage lung cancer screening action in rural eligible populations

**DOI:** 10.1016/j.pecinn.2024.100298

**Published:** 2024-06-01

**Authors:** Dannell Boatman, Lauren McCauley-Hixenbaugh, Abby Starkey, Amy Allen, Stephenie Kennedy-Rea

**Affiliations:** a West Virginia University School of Medicine, Department of Cancer Prevention & Control, USA; b West Virginia University Cancer Institute, USA

**Keywords:** Lung cancer, Lung cancer screening, Health communication, Health belief model, Extended parallel process model, Health campaigns, Rural, Appalachia

## Abstract

**Objective:**

The purpose of this study was to develop an effective communication approach to encourage lung cancer screening action within rural screening-eligible populations.

**Methods:**

An iterative research approach using targeted engagement with the priority population was used. Findings were triangulated through multiple methods, including two surveys and concept testing interviews. The Health Belief Model and the Extended Parallel Process Model served as study frameworks.

**Results:**

Initial findings suggest that threat levels are high in the priority population and an emphasis on barrier mitigation messaging may drive action. Health campaign posters integrating these findings were developed and tested with the priority population. The new health campaign posters were tested against examples of previously used health campaign posters. Findings suggest that the new health campaign posters were more effective in spurring lung cancer screening motivation and intention to act in the priority population compared to current health campaign poster examples.

**Conclusion:**

Messaging focused on gain-framing, inoculation messaging, and barrier mitigation may be more effective in encouraging lung cancer screening action in rural eligible populations.

**Innovation:**

This project outlines a systematic process to developing effective, targeted communication approaches using behavior change and persuasive communication frameworks along with engagement from priority populations.

## Highlights

• Lung cancer screening messaging usually focuses on smoking and fear appeals.
• Threat in screening eligible populations is already elevated.
• Increasing self-efficacy to overcome screening barriers is key.
• Messaging that separates smoking from screening can counter patient arguments.
• Gain-framing of messages can balance heightened fear with response efficacy.

## 1 Introduction

Lung cancer is the leading cause of cancer-related deaths in the United States (US) for both men and women [1]. Most lung cancer cases are diagnosed at a late stage, contributing to poor health outcomes [1]. Early detection of lung cancer is critical for better prognoses [2]. Tobacco use has been identified as the primary cause of lung cancer [3]. People who smoke are 15 to 30 times more likely to die from lung cancer compared to those who do not [1]. The United States Preventive Services Task Force recommends annual lung cancer screening (LCS) using low dose computed tomography for people aged 50–80 years with a significant smoking history (past or present) [4]. Despite this recommendation, LCS rates in the US remain low [5]. Screening eligible individuals may be disinclined to follow LCS recommendations due to the perceived stigma associated with tobacco use, the addictive nature of nicotine, and overall medical mistrust [5].

Lung cancer mortality is even greater in rural areas, such as the US Appalachian region. Appalachia has one of the highest smoking prevalence rates in the country [[6], [7], [8]]. Lung cancer mortality rates are 15%–36% higher in Appalachian counties, and regional incidence rates are the highest in the nation [[6], [7], [8], [9]]. LCS rates in Appalachian counties are even lower than the already suboptimal national average [6]. Structural (e.g., healthcare access limitations, transportation challenges) and psychosocial (e.g., increased medical mistrust, lower literacy levels) factors have been identified as contributors to these regional lung cancer disparities [9]. Due to the significant burden in Appalachia, identifying targeted strategies to improve LCS rates in this region are needed [10].

Consistent messages targeted to specific populations can encourage LCS uptake [11]. While themes such as family, hope, and prolonged life have been shown to resonate with rural LCS-eligible populations [10,11], these concepts have not been aligned with health behavior and persuasive communication frameworks. The linkage between theory and communication content is essential to developing messaging that can move individuals toward positive health behavior action (e.g., conversations with providers, screening). Existing LCS health campaigns and messaging focuses heavily on “de-normalizing” tobacco use through fear appeals and imagery which may be stigmatizing to those at an increased risk for lung cancer [12]. There are mixed findings as to the effectiveness of this approach [12] and no identified studies assessing the impact of this type of messaging in a rural Appalachian population. While overall public awareness has increased through the use of health campaigns, the stigma associated with a lung cancer diagnosis remains high and may contribute to low screening rates [13]. Furthermore, the effectiveness of different framing approaches (gain versus loss) in prompting health behavior action is inconsistent and inconclusive [14] suggesting ambiguity on how to best present LCS messages to high-risk populations.

### 1.1 Study Frameworks

The Health Belief Model (HBM) is a framework used to understand individual behavior change through the use of six constructs: risk susceptibility, risk severity, benefits to action, barriers to action, self-efficacy, and cues to action [15]. The premise is that behavior change will occur if these constructs are successfully addressed during an intervention [15]. The HBM, despite its limitations, has been identified as an ideal framework for health promotion and health communication research and has been used to develop messaging for a variety of mediums [15].

The Extended Parallel Process Model (EPPM) is one of the most common frameworks used to examine fear in persuasion [16]. It is a useful model for health communication professionals as it can guide decision-making for campaign development [16]. The premise of the EPPM is that when exposed to a risky health situation, individuals go through two cognitive appraisals, one focused on perceived efficacy (self and response) and the other on perceived threat (susceptibility and severity) [17]. Response to risk (e.g., getting screened or not) is influenced by this cognitive appraisal.

### 1.2 Study Purpose & Aims

The purpose of this study was to develop the most effective communication approach to encourage LCS action within the rural Appalachian screening eligible population. Following best practices in developing health communication interventions, an iterative approach with targeted engagement of the priority population was used [18]. Study aims were to:

1) Administer a cross-sectional survey (*exploratory survey*) to understand existing LCS perspectives and messaging preferences of LCS eligible Appalachians using the HBM framework;
2) Develop health campaign posters using a new communication approach derived from exploratory survey findings and informed by the EPPM and feedback from screening eligible Appalachians (*concept testing interviews*); and
3) Conduct a cross-sectional survey (*intention survey*) to preliminarily assess the effectiveness of the health campaign posters developed using the new communication approach compared to the current communication approach.

## 2 Methods

### 2.1 Ethics

This study was exempted by the West Virginia University Institutional Review Board (protocol # 2203544332).

### 2.2 Participants

Individuals were eligible for participation in the exploratory survey, key informant interviews, or intention survey if they were West Virginia (WV) residents over the age of 50 years. WV was selected as the study location as it is the only state located completely within the Appalachian region, which was the priority population for this research. Individuals with a smoking history and those without a smoking history were recruited for the exploratory study to understand potential differences based on smoking status. Participants in the key informant interviews and intention survey focused on LCS eligible Appalachians. No incentives were provided for participation.

### 2.3 Exploratory Survey Procedures & Analysis

An exploratory survey was used to understand the existing LCS perspectives and messaging preferences of screening eligible Appalachians using the HBM framework. Survey instrumentation included four main sections: demographics, Champion's HBM Scale [15], messaging preferences by HBM constructs, and message framing preferences. Champion's HBM scale [15] was adapted to assess for LCS perspectives by construct (e.g., threat, benefit, barriers, cues to action). In addition, ten pre-constructed messages were aligned with HBM constructs and presented to participants to understand LCS message preferences. Messages were selected from existing examples of text used for public health campaigns from various national and regional organizations. These messages were identified via an internet search of “lung cancer screening social media messages” and “lung cancer screening awareness campaigns.” The research team reached a consensus on the alignment of HBM constructs with messages selected for survey inclusion. For each message, participants were asked if the content would encourage them to consider LCS. Finally, participants were asked questions related to gain and loss framing of similar messages [14] included in the survey to understand preferences.

The exploratory survey was reviewed by three public health experts for content and piloted with ten individuals prior to dissemination. Participants were recruited from March to November 2022 from a variety of venues, including health fairs, social media, primary care clinics, and using a mobile LCS unit that travels WV. Based on pilot study sample sizes [19], the research team recruited 120 participants using a convenience sampling strategy.

During data cleaning, participant smoking status was coded as “currently (smokes),” “formerly (smoked),” and “never (smoked)” and served as the primary independent variable for analyses. In addition, a new grouping variable was created by combining participants with a “currently (smokes)” and “formerly (smoked)” smoking status into a “smoking history” group. Subscale variables were computed based on items aligned with specific constructs for the adapted Champion HBM scale and the HBM construct-aligned messages. Questions related to susceptibility and severity were combined to compute a new variable, entitled “threat” [15,20]. Between group differences by smoking status were run for each subscale. Champion's HBM scale questions were answered using a 5-point Likert response scale ranging from 1.00 (*strongly disagree*) to 5.00 (*strongly agree)*, and mean scores were computed for each subscale. Higher mean scores represented perceptions of greater threat of lung cancer, greater benefits from screening, greater motivation to screen, and increased barriers to screening. Higher mean scores for threat, cues to action, and benefit subscales have been shown to positively correlate with screening uptake [20]. The opposite has been shown for higher mean barrier scores [20]. HBM construct-aligned message responses were coded “No/Unsure” (1.00) and “Yes” (2.00). Mean scores were computed for HBM construct subscales. Higher scores would suggest greater impact of specific HBM construct messages on participants (e.g., message preference). Questions about gain/loss framing preferences were coded “gain framed preference” (1.00) and “loss framed preference” (2.00). Descriptive statistics were used to characterize framing preferences between groups.

### 2.4 Concept Testing Procedures & Analysis

The research team developed a set of three health campaign poster drafts targeted to the rural Appalachian screening eligible population based on key findings from the exploratory survey viewed through the lens of the EPPM. Based on the best practices in health communication intervention development [18], members of the priority population (*n* = 15) were shown these drafts and asked for feedback on visual imagery, text, and overall concept. The concept testing processes were informed by best practices in health communication program development [21]. Participants were recruited from January to April 2023 from a variety of venues, including health fairs, social media, and primary care clinics using a convenience sampling strategy.

### 2.5 Intention Survey Procedures & Analysis

A set of three health campaign posters representing the new communication approach developed by the research team were finalized through the concept testing process. To preliminary test the effectiveness of this new communication approach compared to the existing communication approach focused on fear and smoking [12], a cross-section survey was used to measure intention and motivation to take LCS action. The research team conducted an internet search for LCS health campaign posters using key terms such as “lung campaign posters,” “lung cancer posters,” and “lung cancer screening posters.” The team reached consensus on a set of four posters from national and regional organizations that represented the predominant messaging approach.

Survey participants viewed seven posters (four current communication approach, three new communication approach) and answered questions related to intention and motivation to talk to their providers about screening and specific questions associated with HBM constructs. For instance, “This poster made me feel like lung cancer is severe” was used to measure perceived severity after viewing the poster. Posters were assigned a random order of appearance in the survey. Each of the seven posters had a total of seven questions, two focused on motivation and intention and five focused on specific HBM constructs. Posters were assessed individually and collectively as part of either the “New Communication Approach” group or the “Current Communication Approach” group. Motivation was assessed using a scale from 0 (not at all motivated) to 10 (extremely motivated). All other responses were coded “No/Unsure” (1.00) and “Yes” (2.00). Higher mean scores represented perceptions of greater threat of lung cancer, greater benefits from screening, greater self-efficacy to overcome screening barriers, and greater intention to take LCS action.

Similar to the exploratory survey, the intention survey was reviewed by three public health experts for content and piloted with ten individuals prior to dissemination. Participants were recruited from July to December 2023 at a variety of venues, including health fairs, social media, primary care clinics, and using a mobile LCS unit. Based on pilot study sample sizes [19], the research team recruited 52 participants using a convenience sampling strategy.

## 3 Results

All statistical analyses were completed using SPSS v28. Descriptive statistics were run to characterize the survey participant population (See Table 1).

**Table 1:** Survey Participant Characteristics.

	Exploratory Survey (*n* = 120)	Intention Survey (*n* = 52)
	*% (n)*	
Average Age	50.0	59.7
Sex		
Female	85.0 (102)	73.1 (38)
Male	14.2 (17)	26.9 (14)
Non-Binary	0.8 (1)	

Race		
Asian	0.8 (1)	
Black	7.5 (9)	1.9 (1)
Mixed Race	1.7 (2)	
White	90.0 (108)	98.1 (51)

Ethnicity		
Hispanic	3.3 (4)	3.8 (2)
Non-Hispanic	96.7 (116)	96.2 (50)

Education Level		
High School or Less	28.3 (34)	44.2 (23)
Some College/Technical School	27.5 (33)	17.3 (9)
College Degree and Higher	44.2 (53)	36.5 (19)

Smoking Status		
Currently Smoking	27.5 (33)	42.3 (22)
Previously Smoked	25.0 (30)	57.7 (30)
Never Smoked	46.7 (56)	

Previous Cancer Diagnosis		
Yes	16.6 (20)	34.6 (18)
No	83.3 (100)	65.4 (34)

### 3.1 Exploratory survey results

A one-way ANOVA was run to assess LCS perspectives by smoking status – “currently (smokes),” “formerly (smoked),” and “never (smoked)” – for the adaptation of Champion's HBM Scale (Table 2). There was a statistically significant difference between smoking status groups for “threat,” *F*(2,109) = 8.7, *p* < .001, with individuals who currently smoke (18.7 ± 3.8) and formerly smoked (18.3 ± 2.8) having more perceived threat than those who had never smoked (16.1 ± 2.9). There was also a statistically significant difference between smoking status groups for “barriers,” *F*(2,111) = 4.6, *p* = .012, with individuals who currently smoke having more perceived barriers to screening (11.8 ± 3.3) compared to individuals who smoked in the past (9.3 ± 3.5) or never smoked (10.8 ± 3.0). There were no statistically significant differences between smoking status groups for the HBM constructs of “benefits” and “cues to action.” An independent *t*-test (Table 2) found that there was a statistically significant difference between participants with a smoking history (both currently smokes and formerly smoked) and those with no smoking history on increased perception of threat (*p* < .001).

**Table 2:** Comparing Lung Cancer Screening Health Belief Model (HBM) Constructs by Smoking Status.

HBM Construct Subscale	Smoking Status	*N*	*M*	*SD*	*t*	*p*
Threat	Smoking History	57	18.5	3.4	4.2	**<0.001**
	Never Smoked	55	16.1	2.9		
	Currently Smoking	29	18.7	3.8		**0.001**
	Never Smoked	55	16.1	2.9		
	Formerly Smoked	28	18.3	2.8		**0.007**
	Never Smoked	55	16.1	2.9		
	Currently Smoking	29	18.7	0.7		0.9
	Formerly Smoked	28	18.3	2.8		
	All Participants	112	17.3	3.3		
Benefits	Smoking History	62	8.9	1.2	0.4	0.7
	Never Smoked	53	8.8	1.7		
	Currently Smoking	33	8.8	1.3		0.8
	Never Smoked	53	8.8	1.7		
	Formerly Smoked	29	9.0	1.1		0.8
	Never Smoked	53	8.8	1.7		
	Currently Smoking	33	8.8	1.3		0.8
	Formerly Smoked	29	9.0	1.1		
	All Participants	115	8.8	1.5		
Barriers	Smoking History	60	10.6	3.6	−0.3	0.8
	Never Smoked	54	10.8	3.0		
	Currently Smoking	31	11.8	3.3		0.3
	Never Smoked	54	10.8	3.0		
	Formerly Smoked	29	9.3	3.5		0.1
	Never Smoked	54	10.8	3.0		
	Currently Smoking	31	11.8	3.3		**0.009**
	Formerly Smoked	29	9.3	3.5		
	All Participants	114	10.7	3.3		
Cues to Action	Smoking History	61	11.8	2.4	0.6	0.6
	Never Smoked	53	11.5	2.2		
	Currently Smoking	33	11.6	2.4		0.9
	Never Smoked	53	11.5	2.2		
	Formerly Smoked	28	11.9	2.4		0.7
	Never Smoked	53	11.5	2.2		
	Currently Smoking	33	11.6	2.4		0.8
	Formerly Smoked	28	11.9	2.4		
	All Participants	114	11.6	2.3		

**Bold** indicates statistical significance.

Independent *t*-tests were run to look at differences between smoking status groups and the HBM-aligned messaging subscales (Table 3). There was a statistically significant difference between smoking status groups and “barrier mitigation” messages, *t*(112) = 2.4, *p* = .009. Participants with a smoking history (both currently smokes and formerly smoked) had a greater preference for barrier mitigation related messages such as “There are lung cancer screening locations around the state,” “The cost of lung cancer screening is covered by Medicaid, Medicare, and most private insurers for those that meet the screening guidelines,” and “It takes less than 10 minutes to complete lung cancer screening” compared to participants without a smoking history.

**Table 3:** Comparing Lung Cancer Screening Message Preferences by HBM Construct and Smoking Status.

Message	HBM Construct	Smoking Status	*N*	*M*	*SD*	*t*	*p*
Even if you quit smoking, you might still be at risk for lung cancer.	Susceptibility (Threat)	Smoking History	61	1.1	0.5	−1.6	0.1
		Never Smoked	55	1.3	0.7		
		Overall	116	1.2	0.6		
If you are a current or former smoker, you are at risk for lung cancer.	Susceptibility (Threat)	Smoking History	62	1.2	0.6	0.4	0.7
		Never Smoked	55	1.1	0.4		
		Overall	117	1.2	0.5		
The best way to prevent lung cancer is not to smoke or quit if you do.	Susceptibility (Threat)	Smoking History	62	1.3	0.6	0.2	0.9
		Never Smoked	55	1.3	0.6		
		Overall	117	1.3	0.6		
Screening can find lung cancer early when it is easier to treat and cure.	Benefits	Smoking History	62	1.2	0.6	1.3	0.2
		Never Smoked	55	1.1	0.4		
		Overall	117	1.1	0.5		
When lung cancer is found early it is treatable.	Benefits	Smoking History	61	1.4	0.8	1.2	0.3
		Never Smoked	55	1.3	0.7		
		Overall	116	1.3	0.7		
It takes less than 10 min to complete lung cancer screening.	Barriers	Smoking History	61	2.1	0.9	1.8	**0.04**
		Never Smoked	53	1.7	0.9		
		Overall	114	1.9	0.9		
Most people will not have to change their clothes.	Barriers	Smoking History	62	2.2	0.9	1.3	0.2
		Never Smoked	53	1.7	0.9		
		Overall	115	2.1	0.9		
The cost of lung cancer screening is covered by Medicaid, Medicare, and most private insurers for those that meet the screening guidelines.	Barriers	Smoking History	62	2.0	0.9	1.9	**0.05**
		Never Smoked	53	1.7	0.9		
		Overall	115	1.8	0.9		
There are lung cancer screening locations around the state.	Barriers	Smoking History	62	2.0	0.9	2.5	**0.01**
		Never Smoked	53	1.6	0.9		
		Overall	115	1.8	0.9		
Lung cancer is the number one cancer killer for men and women.	Severity (Threat)	Smoking History	62	1.8	0.9	0.6	0.6
		Never Smoked	53	1.7	0.9		
		Overall	115	1.8	0.9		
Lung cancer kills more West Virginians than breast, prostate, and colorectal cancer combined.	Severity (Threat)	Smoking History	62	2.0	0.9	1.4	0.2
		Never Smoked	52	1.7	0.9		
		Overall	114	1.8	0.9		
**HBM Construct Subscales**		**Smoking Status**	***N***	***M***	***SD***	***t***	***p***
Threats		Smoking History	61	7.3	2.5	0.52	0.6
		Never Smoked	52	7.1	2.2		
		Overall	113	7.2	2.4		
Benefits		Smoking History	61	2.6	1.2	1.4	0.2
		Never Smoked	55	2.3	0.9		
		Overall	116	2.5	1.1		
Barriers		Smoking History	61	8.3	3.2	2.4	**0.02**
		Never Smoked	53	7.0	2.6		
		Overall	114	7.7	3.0		

**Bold** indicates statistical significance.

Frequencies were run to assess for message framing preferences (gain framed or loss framed) for similar content. Both participants with a smoking history and without a smoking history preferred gain framed messages in 60% of their selected choices, which was not a statistically significant difference between the groups.

### 3.2 Concept testing results

**Fig. 1:**
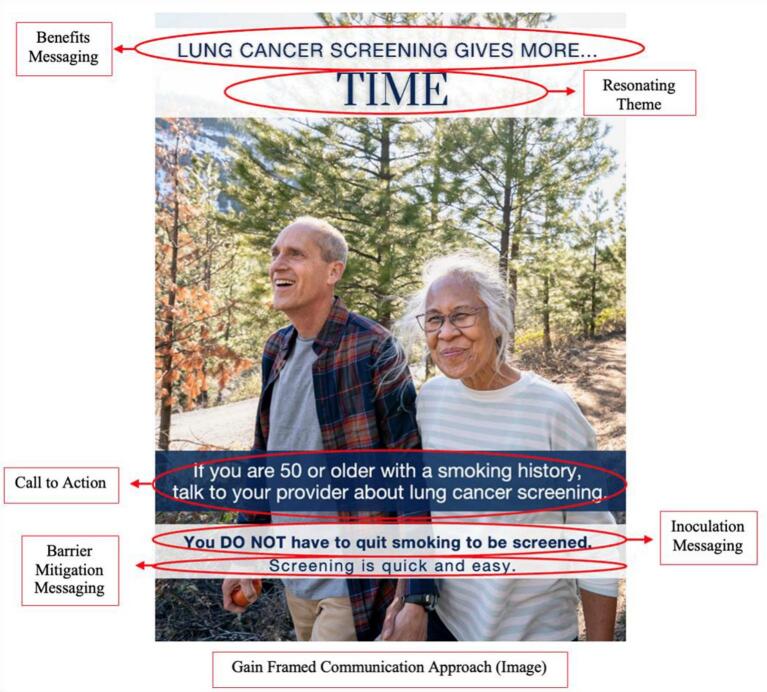
Integration of exploratory survey findings into health campaign poster design.

The research team conceptualized a set of three health campaign posters based on findings from the exploratory survey and framed them using the EPPM (Fig. 1). Key elements of these posters included: 1) gain framing to balance elevated threat with response efficacy, 2) inoculation messaging to counter potential psychological reactance, and 3) increasing self-efficacy through barrier mitigation. Gain framing was selected as individuals with a smoking history selected gain framed messages more frequently than loss framed messages. Through the lens of EPPM, threat was found to be elevated in individuals with a smoking history while barriers were higher with individuals currently smoking. This heightened perception of barriers was not present with individuals who had previously quit smoking, suggesting that smoking was a key difference in this construct. Elevated perception of threat coupled with a high number of reported barriers suggests the potential for psychological reactance which could contribute to counter-intuitive actions or inertia for this high-risk group [22]. To that end, the research team integrated inoculation messaging strategies to address the potential for psychological reactance in individuals who currently smoke [23]. Finally, the “barrier” construct was identified several times as a critical element for individuals with a smoking history when considering LCS so messaging to address key themes such as cost, ease, and convenience were integrated into the health campaign poster design. Concept testing interviews (*n* = 15) were conducted to inform the health campaign poster drafts. Overall, the concept and wording was well received by participants. Feedback from the key informants focused on selecting people who looked like “neighbors” and selecting relatable hobbies, such as fishing and hiking.

#### 3.2.1 Intention Survey Results

**Fig. 2:**
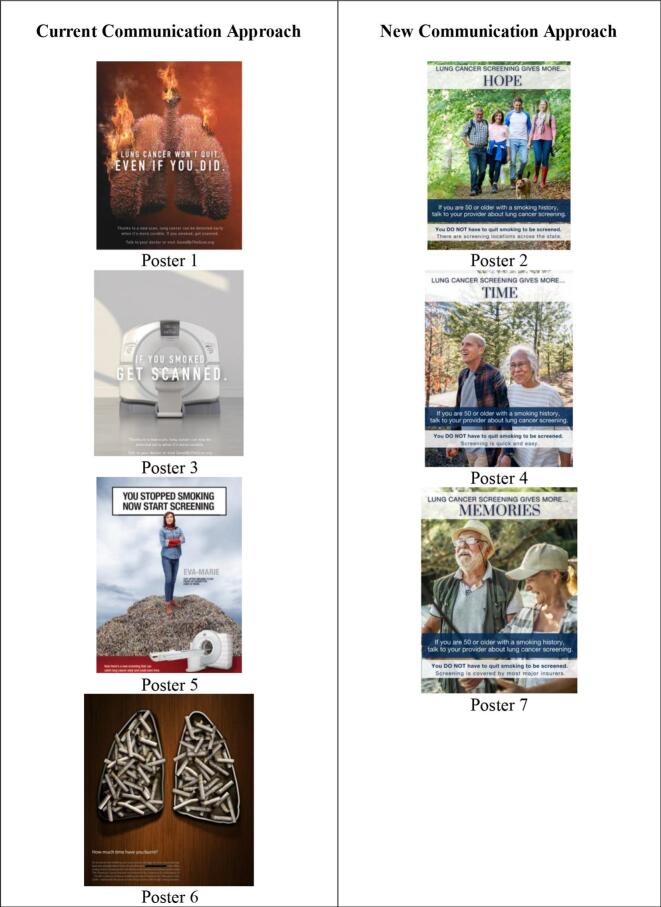
Health campaign posters used for the intention survey grouped by approach.

After the concept testing process, the research team finalized a set of three health campaign posters using the new communication approach and tested them for preliminary effectiveness in increasing motivation and intention to take LCS action compared to a set of four health campaign posters using current communication approaches (Fig. 2). Paired *t*-tests were used to look at differences by HBM construct along with motivation and intention for both “New Communication Approach” group posters and the “Current Communication Approach” group posters (Table 4). Participants with a smoking history were more motivated to talk with their providers after reviewing “New Communication Approach” group posters (6.8 ± 2.6) as opposed to “Current Communication Approach” group posters (5.9 ± 2.8), a statistically significant difference, *t*(51) = −2.6, *p* = .01. Participants had an increased intention to screen (*p* = .007) and improved self-efficacy to overcome screening barriers (*p* = .04) after viewing the “New Communication Approach” group posters compared to the “Current Communication Approach” group posters.

**Table 4:** Comparing Lung Cancer Screening Communication Approach Preferences by Health Belief Model (HBM) Construct/Action Item for Participants with a Smoking History.

HBM Construct Action Item	Messaging Approach	*N*	*M*	*SD*	*t*	*p*
Motivation	Current New	52	5.9	2.8	−2.6	**0.01**
			6.8	2.6		
Intention	Current New	52	1.6	0.3	−2.8	**0.007**
			1.7	0.4		
Severity	Current New	52	1.7	0.3	−1.9	0.07
			1.6	0.4		
Susceptibility	Current New	52	1.8	0.3	0.3	0.7
			1.8	0.4		
Benefits	Current New	52	1.8	0.3	1.0	0.3
			1.8	0.3		
Barriers	Current New	52	1.6	0.4	−2.1	**0.04**
			1.7	0.4		
Cues to Action	Current New	52	1.7	0.3	1.4	0.1
			1.8	0.4		

**Bold** indicates statistical significance.

## 4 Discussion and Conclusion

Findings indicate that study participants with a smoking history understand that they are at risk for lung cancer, and that lung cancer is severe. The messages that screening eligible Appalachians indicate would drive them toward LCS action focused on the test itself, not smoking or threat. This differs from the direction of many national health advertising campaigns that focus on “de-normalizing” tobacco use with fear appeals [12]. Understanding the nuance between improving knowledge and efficacy but not focusing solely on threat escalation which can foster stigma may be important to developing more effective LCS messaging in rural areas like Appalachia. Addressing HBM constructs outside of “susceptibility” and “severity” (“threat”) may be important to reach rural individuals within this screening eligible group.

In the exploratory survey, participants who reported currently smoking indicated a higher level of perceived barriers to LCS, suggesting this is a particularly important construct to consider for those at a high risk for lung cancer. An increased perception of threat and barriers to screening suggests the potential for psychological reactance [22]. Specific themes related to convenience and ease of screening resonated with participants with a smoking history. Strategies to support self-efficacy throughout the screening process could be an important intervention to consider when working with individuals that currently smoke to reduce the impact of perceived barriers and encourage action. These insights could also provide important guidance to reaching other rural populations characterized by lower socioeconomic levels and increased medical mistrust.

The “New Communication Approach” group posters focused on three key findings based on the exploratory survey findings and informed by the EPPM: 1) gain framing to balance elevated threat with response efficacy, 2) inoculation messaging to counter potential psychological reactance, and 3) increasing self-efficacy through barrier mitigation. The “Current Communication Approach” group posters focused on the predominant strategies of 1) loss framing, 2) smoking and smoking cessation, and 3) fear and threat. The “New Communication Approach” drove potential action in screening eligible participants, measured as intention and motivation, at statistically significant higher rates compared to the “Current Communication Approach” group posters. Matching the exploratory study findings, focusing on developing self-efficacy to overcome barriers to screening may be key to prompting action in rural screening eligible populations.

Study findings suggest that current messaging approaches may not effectively drive LCS action in the rural Appalachian screening eligible populations. This poses a challenge to public health efforts as lung cancer burden is significant across the US and particularly within rural hotspots like Appalachia. These findings also suggest the importance of audience segmentation in health message development. Overall, within the rural screening eligible population in Appalachia, communication strategies that can drive LCS action should focus on 1) gain framing to balance elevated threat with response efficacy, 2) inoculation messaging to counter potential psychological reactance, and 3) increasing self-efficacy through barrier mitigation. This is complementary to previous research in the region that highlighted resonating themes such as hope, prolonged, life, and family [10,11].

### 4.1 Innovation

This project is innovative for multiple reasons, including both research findings and approach. Through this study, a new communication approach was developed that was found to be more effective in spurring LCS action in rural Appalachian screening eligible populations. Current LCS communication approaches are closely aligned with smoking cessation. This new communication approach adds separation between smoking cessation messaging and LCS messaging to counter any potential psychological reactance for individuals currently smoking and who may not be ready to quit. While conversations about smoking cessation have been shown to be potentially successful at the point of LCS service [24], the initial exposure to messages separating the two may provide a sense of personal choice in the situation through inoculation messaging strategies. While recent research has highlighted the benefits of gain framed messages to prompt LCS in eligible populations [25], there are few prominent health campaigns that use this approach. Finally, using a health behavior change framework was integral to identifying a key construct that was critical to prompting LCS action within this priority population. By isolating a key element associated with action, barrier mitigation, and effective messaging could be integrated. Taken collectively, this is an innovative approach to LCS communication and messaging.

This study also emphasizes the importance of iterative health communication research to ensure that the right messages reach the right audiences to spur targeted action [18]. It is innovative as the study outlines a systematic process to developing effective, targeted communication approaches using both behavior change and persuasive communication frameworks along with engagement from priority populations. This approach could be adapted to other health conditions and populations.

### 4.2 Limitations

This study has limitations related to both methods and recruitment challenges. Surveys and interviews had small, region-specific convenience samples and relied on unweighted data in analysis, so the results are not generalizable. Most study participants were women which also affects generalizability. While this must be taken into consideration when reviewing the findings, women are often the healthcare decision makers within their families [26] and their opinion is critical to understanding preventive service utilization. In addition, recruitment was challenging, highlighting the potential role of stigma in all aspects of the lung cancer conversation [13].

### 4.3 Future Directions

Assessment of the new communication approach should move beyond intention and motivation to documented action. Exploration of this messaging within the clinical space may prove an avenue to look at effects on LCS rates. While testing of intention and motivation focused on the use of health campaign posters, translating these findings to other mediums will be essential to future dissemination efforts. In addition, crafting communication strategies to support more effective interpersonal conversations to encourage LCS action for eligible rural individuals using these approaches should be considered (e.g., messaging approaches targeted to healthcare providers, friends, and family).

## CRediT authorship contribution statement

**Dannell Boatman:** Writing – original draft, Supervision, Project administration, Methodology, Conceptualization. **Lauren McCauley-Hixenbaugh:** Writing – review & editing, Investigation, Conceptualization. **Abby Starkey:** Writing – review & editing, Visualization, Investigation, Conceptualization. **Amy Allen:** Writing – review & editing, Conceptualization. **Stephenie Kennedy-Rea:** Writing – review & editing, Conceptualization.

## Declaration of competing interest

The authors declare that they have no known competing financial interests or personal relationships that could have appeared to influence the work reported in this paper.
